# Supplementary material for: The Systems Biology Research Tool: evolvable open-source software
Source: BMC Syst Biol. 2008 Jun 29;2:55. doi: 10.1186/1752-0509-2-55 (PMC2446383; doi:10.1186/1752-0509-2-55)
Supplement: Additional file 1 — SBRT Archive. An archive of the current version of the Systems Biology Research Tool. [file 1752-0509-2-55-S1.zip › sbrt-1.4.0/doc/developers_guide/api/index-files/index-12.html]

L-Index


|  |  |  |
| --- | --- | --- |
| **Overview** \| Package \| Class \| Use \| **Tree** \| **Deprecated** \| **Index** \| **Help** | |  |
| **PREV LETTER**   **NEXT LETTER** | **FRAMES**    **NO FRAMES**     **All Classes** |  |


A B C D E F G H I J K L M N O P Q R S T U V W X Y Z 

---


## **L**

**lastIndexOf(Object)** - Method in class sbrt.kernel.util.UnmodifiableList: See List.lastIndexOf(Object). **LEFT\_BRACE** - Static variable in class sbrt.shell.text.SpecialChars: { **LEFT\_BRACKET** - Static variable in class sbrt.shell.text.SpecialChars: [ **LEFT\_PAREN** - Static variable in class sbrt.shell.text.SpecialChars: ( **LESS\_THAN** - Static variable in class sbrt.shell.text.SpecialChars: < **LinCombTermFormatV1**<V> - Class in sbrt.shell.text.math: This class is a concrete implementation of `LinearCombTermFormat`. **LinCombTermFormatV1(SimpleFormat<V>)** - Constructor for class sbrt.shell.text.math.LinCombTermFormatV1: Constructs a new linear combination term format using the provided formats. **LinCombTermFormatV2**<V> - Class in sbrt.shell.text.math: This class is a concrete implementation of `LinearCombTermFormat`. **LinCombTermFormatV2(SimpleFormat<V>)** - Constructor for class sbrt.shell.text.math.LinCombTermFormatV2: Constructs a new linear combination term format using the provided formats. **LinearComb**<V> - Interface in sbrt.kernel.math: This interface is used to represent linear combinations. **LinearComb.Term**<V> - Interface in sbrt.kernel.math: This interface is used to represent the terms of linear combinations. **LinearCombConverterManager** - Class in sbrt.shell.mng.algebra: This class is used to convert linear combinations to matrices. **LinearCombConverterManager()** - Constructor for class sbrt.shell.mng.algebra.LinearCombConverterManager: **LinearCombFormat**<V> - Interface in sbrt.shell.text.math: This interface is used to represent formats for linear combinations. **LinearCombFormatV1**<V> - Class in sbrt.shell.text.math: This class is a concrete implementation of `LinearCombFormat`. **LinearCombFormatV1(SimpleFormat<V>, double)** - Constructor for class sbrt.shell.text.math.LinearCombFormatV1: Constructs a new linear combination format using the provided arguments. **LinearCombFormatV1(SimpleFormat<V>)** - Constructor for class sbrt.shell.text.math.LinearCombFormatV1: Constructs a new linear combination format using the provided format. **LinearCombFormatV2**<V> - Class in sbrt.shell.text.math: This class is a concrete implementation of `LinearCombFormat`. **LinearCombFormatV2(SimpleFormat<V>, double)** - Constructor for class sbrt.shell.text.math.LinearCombFormatV2: Constructs a new linear combination format using the provided arguments. **LinearCombFormatV2(SimpleFormat<V>)** - Constructor for class sbrt.shell.text.math.LinearCombFormatV2: Constructs a new linear combination format using the provided format. **LinearCombTermFormat**<V> - Interface in sbrt.shell.text.math: This interface is used to represent the formats of terms from linear combinations. **LinearProgramSolver**<R,C> - Interface in sbrt.kernel.math.optimization: This interface is used to represent linear program solvers. **LinearSolverManager** - Class in sbrt.shell.mng.algebra: This class is used to manage linear system solvers. **LinearSolverManager()** - Constructor for class sbrt.shell.mng.algebra.LinearSolverManager: **LinearSolverProcess**<V> - Class in sbrt.kernel.math.algebra.proc: This class is used to solve systems of linear equations. **LinearSolverProcess()** - Constructor for class sbrt.kernel.math.algebra.proc.LinearSolverProcess: **LinearSystemSolution**<V> - Interface in sbrt.kernel.math.algebra: This interface is used to represent a general solution to a system of linear equations. **LinearSystemSolver**<V> - Interface in sbrt.kernel.math.algebra: This interface is used to represent classes that solve systems of linear equations. **lineIterator()** - Method in class sbrt.shell.io.AbstractMapInputFile: Returns a plain text line iterator over this text file. **lineIterator()** - Method in class sbrt.shell.io.TextInputFile: Returns a line iterator over this text file. **LinkedHashSetFormat**<E> - Interface in sbrt.shell.text: This interface is used to represent `LinkedHashSet` formats. **ListFormat**<E> - Interface in sbrt.shell.text: This interface is used to represent list formats. **listIterator()** - Method in class sbrt.kernel.util.UnmodifiableList: See List.listIterator(). **listIterator(int)** - Method in class sbrt.kernel.util.UnmodifiableList: See List.listIterator(int). **ListResultsManager**<R> - Class in sbrt.kernel.io: This class is used to store a list of results that can be easily retrieved. **ListResultsManager()** - Constructor for class sbrt.kernel.io.ListResultsManager: Constructs a new list-based results manager. **lock()** - Static method in class sbrt.ext.R.R: Locks the R engine so that no one can use it without the key. **LongFormat** - Interface in sbrt.shell.text: This interface is used to represent a format for longs. **LongFormatV1** - Class in sbrt.shell.text: This class is a concrete implementation of `LongFormat`. **LongFormatV1()** - Constructor for class sbrt.shell.text.LongFormatV1: **lowerBoundsEqualZero(Map<?, Interval>, double)** - Method in class sbrt.kernel.fba.analysis.FluxIntervalAnalyzer: Returns the number of provided intervals whose lower bound equals zero. **lowerBoundsEqualZero(Collection<Interval>, double)** - Method in class sbrt.kernel.fba.analysis.FluxIntervalAnalyzer: Returns the number of provided intervals whose lower bound equals zero. **lowerBoundsGreaterThanZero(Map<?, Interval>, double)** - Method in class sbrt.kernel.fba.analysis.FluxIntervalAnalyzer: Returns the number of provided intervals whose lower bounds are greater than zero. **lowerBoundsGreaterThanZero(Collection<Interval>, double)** - Method in class sbrt.kernel.fba.analysis.FluxIntervalAnalyzer: Returns the number of provided intervals whose lower bounds are greater than zero.

---


|  |  |  |
| --- | --- | --- |
| **Overview** \| Package \| Class \| Use \| **Tree** \| **Deprecated** \| **Index** \| **Help** | |  |
| **PREV LETTER**   **NEXT LETTER** | **FRAMES**    **NO FRAMES**     **All Classes** |  |


A B C D E F G H I J K L M N O P Q R S T U V W X Y Z 

---
